# Phylogeny, expression patterns and regulation of DNA Methyltransferases in early development of the flatfish, *Solea senegalensis*

**DOI:** 10.1186/s12861-017-0154-0

**Published:** 2017-07-17

**Authors:** Joana Firmino, Carlos Carballo, Paula Armesto, Marco A. Campinho, Deborah M. Power, Manuel Manchado

**Affiliations:** 1 IFAPA Centro El Toruño, Junta de Andalucía, Camino Tiro Pichón s/n, 11500 El Puerto de Santa María, Cádiz, Spain; 2 Comparative Molecular Endocrinology Group, Marine Science Centre (CCMAR), Universidade do Algarve, 8005-139 Faro, Portugal

**Keywords:** *Solea senegalensis*, DNA methyltransferases, Thermal programming, Gene expression

## Abstract

**Background:**

The identification of DNA methyltransferases (Dnmt) expression patterns during development and their regulation is important to understand the epigenetic mechanisms that modulate larval plasticity in marine fish. In this study, *dnmt1* and *dnmt3* paralogs were identified in the flatfish *Solea senegalensis* and expression patterns in early developmental stages and juveniles were determined. Additionally, the regulation of Dnmt transcription by a specific inhibitor (5-aza-2′-deoxycytidine) and temperature was evaluated.

**Results:**

Five paralog genes of *dnmt3*, namely *dnmt3aa*, *dnmt3ab, dnmt3ba*, *dnmt3bb.1* and *dnmt3bb.*2 and one gene for *dnmt1* were identified. Phylogenetic analysis revealed that the *dnmt* gene family was highly conserved in teleosts and three fish-specific genes, *dnmt3aa*, *dnmt3ba* and *dnmt3bb.2* have evolved*.* The spatio-temporal expression patterns of four *dnmts* (*dnmt1*, *dnmt3aa*, *dnmt3ab* and *dnmt3bb.1*) were different in early larval stages although all of them reduced expression with the age and were detected in neural organs and *dnmt3aa* appeared specific to somites. In juveniles, the four *dnmt* genes were expressed in brain and hematopoietic tissues such as kidney, spleen and gills. Treatment of sole embryos with 5-aza-2′-deoxycytidine down-regulated *dntm1* and up-regulated *dntm3aa*. Moreover, in lecithotrophic larval stages, *dnmt3aa* and *dnmt3ab* were temperature sensitive and their expression was higher in larvae incubated at 16 °C relative to 20 °C.

**Conclusion:**

Five *dnmt3* and one *dnmt1* paralog were identified in sole and their distinct developmental and tissue-specific expression patterns indicate that they may have different roles during development. The inhibitor 5-aza-2′-deoxycytidine modified the transcript abundance of *dntm1* and *dntm3aa* in embryos, which suggests that a regulatory feedback mechanism exists for these genes. The impact of thermal regime on expression levels of *dnmt3aa* and *dnmt3ab* in lecithotrophic larval stages suggests that these paralogs might be involved in thermal programing.

**Electronic supplementary material:**

The online version of this article (doi:10.1186/s12861-017-0154-0) contains supplementary material, which is available to authorized users.

## Background

DNA methylation is a key regulatory mechanism during embryonic development. Methylation normally occurs at CpG dinucleotides and modulates transcription cascades, genomic imprinting as well as genome stability and chromatin structure [1–7]. DNA Methylation is catalyzed by DNA methyltransferases (Dnmt) of the Dnmt1 and Dnmt3 families. In mammals, Dnmt1 is the most abundant enzyme and acts mainly as a maintenance methyltransferase to ensure the inheritance of methylation patterns during cell division [8] and it has a direct role on histone methylation [9, 10]. The Dnmt3 enzymes are more diverse (with three genes in mammals Dnmt3a, Dnmt3b and Dnmt3L) and are responsible for de novo DNA methylation in germ cells and in preimplantation embryos [8, 11, 12]. De novo methylation is an essential mechanism of plant and animal development, particularly during tissue differentiation when cells undergo epigenetic reprogramming to adopt a new phenotype [13, 14]. The identification of well-conserved Dnmt1 and Dnmt3 proteins in vertebrates including teleosts suggests that these regulatory pathways may be conserved across vertebrates [15]. However, the teleost specific whole genome duplication has increased the *dnmt* copy number and six *dnmt3* paralogs have been identified in *Danio rerio* [15–17]. The consequences during development of the extra gene copies of *dnmt* in fish remains to be established.

Although some environmental conditions such as oxygen levels, diets or lighting can modulate DNA methylation patterns, temperature acts as a major epigenetic regulator in embryonic and larval stages of fish [18–20]. For example, higher global methylation levels of genomic DNA occur in fish from polar regions relative to those from tropical and temperate regions [21]. Moreover, temperature regime during early development in fish induces epigenetic reprogramming modifying muscle fibre formation and composition, which affects somatic growth in adults [18, 22]. Thermally-dependent programming of muscle growth has been reported in *Salmo salar* [23, 24], *Gadus morhua* [25], *Dicentrarchus labrax* [26, 27], *Oncorhynchus mykiss* [26], and *D. rerio* [28]. Recent studies have demonstrated that some of the effects on muscle of thermal programming are linked to changes in the methylation pattern of genes involved in myogenesis [29] establishing DNA methylation as a key regulatory mechanism of muscle development. Moreover, temperature can also affect sex differentiation by modifying methylation patterns of genes that are sexually dimorphic and by influencing endocrine regulation [7, 30–33]. Thus, the study of Dnmt genes and their regulation by environmental factors may be critical to understand and monitor epigenetic modulation of traits of high relevance for aquaculture.

The Senegalese sole is a flatfish of high interest for aquaculture. The physiological and morphological changes that drive the transition to a benthic mode of life occur early during development (12–20 days post-hatch) and are sensitive to environmental and nutritional factors [34]. Previous studies in sole demonstrated that different temperature regimes during the pelagic larval stage greatly influenced the growth trajectory of post-larval stages [35–37]. When premetamorphic larvae were reared at low temperatures (~15 °C), a drop in their digestive capacity occurred and protein metabolism was modified [35]. Muscle cellularity and the expression of genes involved in muscle development such as myogenin (*myog*) decreased as a consequence of increased promoter methylation [36, 37]. The differential expression of *dnmt1* and *dnmt3* genes was suggested to be essential for this modulation [37]. Moreover, modified development and skeletal muscle cellularity caused by the temperature regime during embryogenesis was also reported [38]. In addition to the effects on muscle, thermal cycles during larval rearing of fish can also change the sex ratio of the population [39]. All these data indicate the importance of methylation in modulating the plasticity of larval development and the involvement of Dnmts requires further study.

To better understand the role of methylation in the epigenetic regulation of Senegalese sole we: (i) Identified Dnmt-encoding genes in *S. senegalensis*; (ii) Characterized spatio-temporal expression patterns of Dnmts during larval development and in juvenile tissues; (iii) Used a methyltransferase inhibitor (5-aza-2′-deoxycytidine) to establish the regulation of *dnmt1*, *dnmt3aa*, *dnmt3ab* and *dnmt3bb.1*; and (iv) Evaluated the short-term effects of temperature regimes during larval lecithotrophic stages on *dnmt* gene expression.

## Methods

### Larval rearing and experimental trials

The samples of juvenile liver, spleen, brain, gills, intestine, head kidney, heart, skeletal muscle, and skin used in this work were obtained in a previously unrelated study [40]. For larval trials, fertilized eggs of Senegalese sole were obtained from CUPIMAR (San Fernando, Cadiz, Spain). Eggs were collected early in the morning (9:00 a.m.) and transferred to a 1000 mL measuring cylinder to separate buoyant (viable) from non-buoyant (non-viable) eggs. The number of eggs in each fraction was estimated using volumetric methods (1100 eggs mL^−1^). Water temperature and salinity in the broodstock tank (20 animals; ratio 2 M:1F) during spawning were 18 °C and 32 ppt, respectively. Breeders were fed with polychaeta, mussels and squid.

To study the regulation of *dnmt* gene expression an inhibitor of Dnmt activity, 5-aza-2′-deoxycytidine (5-AzaCdR, Sigma Aldrich, Spain) was used. A stock of 5-AzaCdR was prepared by dissolving it in 0.01% DMSO to a final concentration of 100 mg/mL; it was stored in aliquots at −20 °C until use. Preliminary trials were performed with 5-AzaCdR and the concentration that did not affect the hatching rates was considered to be the optimal treatment dose. Embryos (in gastrula stage) were randomly distributed between fifteen 50 mL sterile-containers (100 embryos per container) and exposed to 10 μM and 50 μM 5-AzaCdR (five replicates per condition) or the vehicle, 0.01% DMSO (control). During 5-AzaCdR exposures, the embryos were incubated at 22 °C for 24 h and 100 rpm in a shaker (INFORS HT Unitron). Larvae were examined at hatching (0 days post-hatch (dph)), fixed in RNA-later for qPCR analysis and stored at −80 °C until use. In addition, some larvae were fixed overnight in 4% paraformaldehyde (PFA) at 4 °C, dehydrated in methanol and stored at −20 °C until used for whole-mount in situ hybridization (WISH) as previously described [41, 42].

To evaluate the effect of temperature on lecithotrophic larval stages (before mouth opening), fertilized eggs in gastrula (50% epiboly stage) were distributed into six 1 L beakers containing 750 ml of sea water (32 ppt) at an initial egg density of 2000 embryos L^−1^. The incubation temperature was set at 16 °C for half of the beakers (INFORS HT Unitron) and 20 °C for the remaining ones (INFORS HT aerotron) although in the latter case the final average temperature recorded was 20.8 °C. Egg incubation was carried out by gently agitating the beakers at 100 rpm and the sea water was replaced daily by filtering the larvae through a 350 μm mesh net taking care not to damage them. Embryonic stages were classified following the guidelines reported for zebrafish in Kimmel, CB, et al. [43] due to the lack of a detailed staging series in sole. Larvae were sampled at different time points and fixed in RNAlater for gene expression analysis by quantitative PCR or 4% PFA for WISH analysis as indicated above. To compare equivalent larval developmental stages in the two thermal treatment groups, cumulative degree-hours (CDH) was used to establish the sampling times. CDH was calculated as follows: [(average treatment temperature - T_0_) × time (in hours)], where T_0_ is the threshold temperature (considered to be 13 °C in *S. senegalensis*) [44] and the average treatment temperatures 16 and 20.8 °C. Moreover, larval growth as determined by total length, total area and yolk sac length were also measured in 25 photographed larvae per condition using ImageJ v1.47 software.

### Sequence and phylogenetic analysis

Sequences encoding Senegalese sole *dnmt1, dnmt3aa, dnmt3ab* and *dnmt3bb.1* were identified after carrying out a search in the databases SoleaDB *v*3 and *v*4.1 [34]. The accession number of each transcript is indicated in Additional file 1. Sequences were downloaded and assembled using Seqman*v*8.1 (Lasergene, DNASTAR) and edited using EditSeq. Multiple sequence alignments of sole *dnmt* and orthologues from other teleosts were performed using clustalW in MegAlign*v*8.1.

**Fig. 1:**
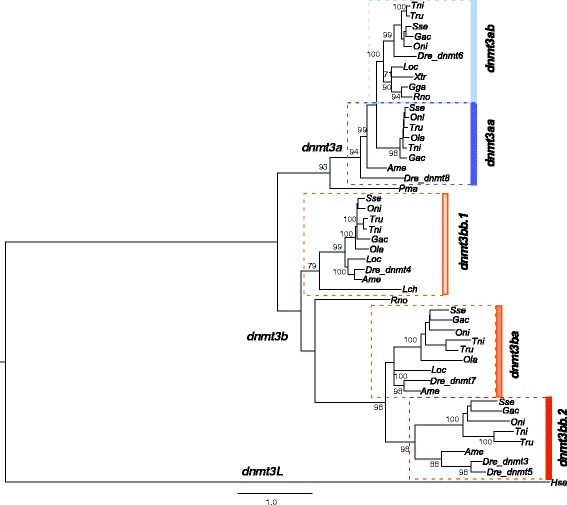
Phylogenetic relationships among the predicted amino acid sequences of *dnmt3* genes in Senegalese sole and other vertebrates (see Additional file 1 for listed names, accession numbers and abbreviations of each species) using the Maximum Likelihood method. The tree was rooted using the *dnmt3L* sequence. Only bootstrap values higher than 70% are indicated on each branch. The scale for branch length (1.0 substitutions/site) is shown below the tree

For phylogenetic analysis, sequences encoding methyltransferases from other vertebrates encoding for methyltransferases were retrieved from the ENSEMBL (www.ensembl.org) databases (Additional file 1). Maximum likelihood (ML) phylogenetic analysis was carried out using ProtTest*v*3.2 and the PHYLIP package. The best-fit model for sequence evolution was JTT + I + G + F. For the complete set of *dnmts*, −lnL was 68,834.14 with a gamma distribution shape parameter (four rate categories) of 0.947 and a proportion of invariable sites of 0.017 (Additional file 2). For the phylogenetic analysis of dnmt3 paralogs (Fig. 1), the lnL was 68,622.37 with a gamma shape of 0.83 and proportion of invariable sites 0.009. The PHYLIP package was then employed to estimate the bootstrap values using SEQBOOT (100 replicates) and the data were analyzed using the software PHYML. The consensus phylogenetic tree was then obtained (CONSENSE). Trees were drawn using Figtree *v*1.4.2 (http://tree.bio.ed.ac.uk/software/figtree/). Synteny analysis was carried out using Genomicus genome browser (http://www.genomicus.biologie.ens.fr/genomicus-79.01/cgi-bin/search.pl).

### RNA isolation and RT-qPCR analysis

Pools of embryos (*n* = 20) and lecithotrophic larvae (*n* = 15) accounting for ~40 mg wet weight were homogenized using a Fast-prep FG120 instrument (Bio101) and Lysing Matrix D (Q-Bio- Gene) for 40s at speed setting 6. The numbers of embryos/larvae in the pools were always similar between conditions and replicates. Total RNA was isolated using an RNeasy Mini Kit (Qiagen). All RNA isolation procedures were carried out in accordance with the manufacturer’s protocol. In all cases, total RNA was treated twice with DNase I using an RNase-Free DNase kit (Qiagen) to eliminate genomic DNA contamination. RNA sample quality was checked by agarose gel electrophoresis, and quantified using a Nanodrop ND-8000 (Thermo Scientific).

Total RNA (1 μg) from each sample was reverse-transcribed using an iScript™ cDNA Synthesis kit (Bio-Rad) and following the manufacturer’s instructions. Real-time analysis was carried out on a CFX96™ Real-Time System (Bio-Rad) using Senegalese sole specific primers for each transcript (Table 1). Real-time reactions were performed in duplicate 10 μl reaction volumes containing cDNA generated from 10 ng of original RNA template, 300 nM each of specific forward and reverse primers, and 5 μl of SYBR Premix Ex Taq (Takara, Clontech). The amplification protocol used was as follows: initial 7 min denaturation and enzyme activation at 95 °C, then 40 cycles of 30s at 95 °C, 15 s at 68 °C and 30s at 72 °C (95 °C, 40 cycles of 95 °C for 15 s and 70 °C for 30s). After template amplification, a melt curve analysis was performed to confirm that a single PCR amplicon was generated. For larval experiments, all data were normalized using the geometric mean of ubiquitin (*ub52*) and beta actin (*actb1*) [45]. For juvenile tissues, 18S rDNA was selected as a suitable reference gene [40, 46]. In embryo experiments, the data were normalized using the geometric mean of 18S rDNA and *actb1*. Reference genes for normalization were selected according to their expression stability within each experiment. To estimate the PCR efficiency, a standard curve was generated for each primer pair based on 10-fold serial dilutions of cDNA transcribed from 100 to 0.01 ng of total RNA. Estimated efficiencies were 2.06, 2.01, 2.04 and 2.08 for *dnmt1*, *dnmt3aa*, *dnmt3ab* and *dnmt3bb.1*, respectively. Relative mRNA expression was determined using the comparative 2^-(∆∆Ct)^ method.

**Table 1:** Primers used for RT-qPCR and probe amplification. For *dntm3aa*, the same forward primer was used in both reactions

Target	Primer	Sequence (5′ → 3′)	Amplicon (bp)	Application
*dnmt1*	F	GCCGTCAAGTCACCGACCGCTCCTAA	132	qPCR
	R	CATTGACAAGATGGTTGGCTGTTTCCCA		
	F	TGCACGTGTTTGCGCCCAGAGCTT	669	WISH
	R	GAGCGCACTCCCTCACACTGACCACT		
*dnmt3aa*	F	CGCCCTCACAGCAACACACAGACCCT	126	qPCR
	R	CGATGACGAAGCCCCGCCCATCC		
	R	CCGCACATGTAGCAGTTCCACGGGTCCTCT	994	WISH
*dnmt3ab*	F	TAGAGCCACCAGAGGAGGAGCGCAAT	86	qPCR
	R	GGGGCGTGTAGGCTGCCGCTT		
	F	CATCATCCACCACCGCCACTGCCAC	774	WISH
	R	TGCCGCTTCTGGCTCCACCCACATC		
*dnmt3bb.1*	F	CGGACTGAACCCACACGCGCTCAACA	93	qPCR
	R	AGGACACGAGGACCACCAACGCACAG		
	F	CGGACTGAACCCACACGCGCTCAACA	963	WISH
	R	AGCAGAACCTCTCGACCACCGCAGCAA		

### Whole-mount in situ hybridization (WISH)

To determine the ontogeny of the expression of the four *dnmt* genes during development, the larval samples (from 0 to 9 dph) obtained from standard larval rearing procedures, previously collected and described in [41] were used. These samples were designated as the control and were used in WISH analyses for comparison with the results of larval reared under different thermal regimes or treated with 5-AzaCdR.

To synthesize WISH probes for *dnmt1, dnmt3aa, dnmt3ab* and *dnmt3bb.1*, they were PCR amplified with targeted gene-specific primers. PCR reactions for WISH probe amplification were carried out using cDNA of a larval pool as template. PCR products were cloned into TOPO-TA vector and identity confirmed by cycle sequencing using a BigDye^®^ Terminator v3.1 kit (Applied Biosystems). Both sense and anti-sense probes were prepared using 20 U T3 or T7 polymerase in transcription buffer (Promega) with 1 μl digoxigenin-RNA labeling mix (Roche Diagnostics, Mannheim, Germany) as described in Campinho et al. [47]. PCR amplification conditions were: 94 °C for 2 min, followed by 30 cycles of 30s at 94 °C, 30s at 60 °C and 90s at 72 °C. Primers were designed using Oligo v6.89 software (Medprobe).

For WISH analyses, samples from chemical or temperature trials were fixed in 4% (wt/vol) paraformaldehyde in 1 × PBS overnight at 4 °C, dehydrated in methanol 100% and conserved at −20 °C until processing. Fixed embryos were depigmented and permeabilized (10 μg/ml proteinase K for 16 min at 37 °C) before being soaked in the digoxigenin-labeled probes as previously described [48]. Excess probe was then removed with successive washes in Hyb(−): 2 × SSC to 2 × SSC + 0.1% Tween-20 (2 × SSCT) and then washed twice for 30 min in 0.2 × SSCT at 68 °C followed by a final last wash in 1 × PBT at room temperature. Samples were pre-incubated for 3 h at RT in MABTr +10% sheep serum (Sigma-Aldrich)/2% Blocking solution (Roche) and then incubated overnight at 4 °C in 1/5000 anti-DIG serum (Roche) diluted in MABTr +10% sheep serum/2% blocking solution. For colour development, samples were washed in MABTr and then incubated in NBT/BCIP for color development. Incubation time was for 60–120 min (depending on the larval stage). For observation and storage, larvae were washed in PBS refixed in 4% PFA/1 × PBS for 20 min, washed in PBT solution and then transferred to glycerol. Twenty animals per experimental condition were used. Digital images were captured using a Leica DFC290 HD digital camera attached to a Leica DMIL LED inverted microscope.

### Statistical analysis

Results are expressed as the mean ± standard deviation (SD). All data were checked for normal distribution with the Kolmogorov–Smirnov test as well as for homogeneity of variance with the Levene′s test and when necessary a log transformation was applied. Significant differences during embryonic development and in juvenile tissues were analyzed using one-way ANOVA. To assess if there were significant differences in gene expression and growth between temperature and embryonic or metamorphic stages, a two-way ANOVA was performed using time and treatment as fixed factors. When significant effects of temperature were identified, single ANOVAs were carried out. Statistical analyses were performed using SPSS *v*21 software (IBM Corp) and Statistix 9 (Analytical Software).

## Results

### Identification and phylogeny of methyltransferases in *S. senegalensis*

A search for sequences encoding sole Dnmts in the SoleaDB [34] resulted in 25 putative transcripts, which corresponded to six unique transcripts that were named, *dnmt1*, *dnmt3aa*, *dnmt3ab*, *dnmt3ba*, *dnmt3bb.1* and *dnmt3bb.2* in agreement with Ensembl nomenclature for orthologues. Amino acid identity between the sole *dntm3a* paralogs was 75.1%. Lower amino acid sequence identity was observed between sole *dnmt3b* paralogs that ranged between 40.2 and 49.6% (Table 2). Amino acid sequence identities between *dnmt1* and *dnmt3* paralogs were in the range of 11.0–13.7%.

**Table 2:** Identities (%) between *dnmt* paralogs in sole

	*dnmt1*	*dnmt3aa*	*dnmt3ab*	*dnmt3bb.1*	*dnmt3ba*	*dnmt3bb.2*
*dnmt1*	100	13.7	12.0	11.8	11.0	11.7
*dnmt3aa*		100	75.1	53.6	45.8	41.4
*dnmt3ab*			100	55.1	41.3	35.6
*dnmt3bb.1*				100	49.6	43.6
*dnmt3ba*					100	40.2
*dnmt3bb.2*						100

Phylogenetic analysis of the five *dnmt3* genes identified two main clusters, referred to as *dntm3a* and *dnmt3b* (Fig. 1). The *3a* cluster contained two well-supported clades named *dnmt3aa* and *dnmt3ab* and the *3b* cluster contained three clades with the *dnmt3bb.2* closer to *dnmt3ba* than *dntm3bb.1*. The *dnmt3ba*, *dnmt3bb.2 and dnmt3aa* were fish-specific lineages and *dnmt3bb.2 and dnmt3aa* were only identified in teleosts. Synteny analysis using the *genomicus* platform revealed that spotted gar (*Lepisosteus oculatus*), a basal ray-finned fish, possessed only one *dnmt3a* gene (identified as *dnmt3aa*) that clustered in the phylogenetic analysis within the *dntm3ab* clade close to tetrapods. The *dnmt3ba* and *dntm3bb.1* were arranged in tandem in the spotted gar genome, an organization that was not conserved in *teleostei* although both paralogs had similar flanking genes (i.e. *nol4l* paralogs). The *dntm3bb.1* and *dntm3bb.2* were in tandem in *teleostei*.

Phylogenetic analysis using both the *dnmt1* and *dnmt3* sequences clustered the teleost and tetrapod orthologues separately at a large distance as expected based upon amino acid sequence identity (Additional file 2 and Table 2). The five *dnmt3* clades remained clearly differentiated in this new analysis.

### Expression patterns of dnmt1, dnmt3aa, dnmt3ab and dnmt3bb.1 during embryonic and larval development

**Fig. 2:**
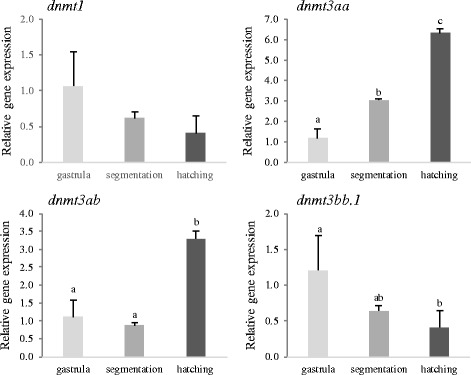
Expression levels of the *dnmt1, dnm3aa, dnmt3ab* and *dnmt3bb.1* at gastrula, segmentation and hatching in embryos incubated at 16 °C and 20 °C. Data are expressed as the mean fold change (mean + SD, *n* = 3) from the calibrator group (gastrula). Different letters denote statistically significant differences among developmental stages for each gene (*P <* 0.05)

Quantification of mRNA levels for *dnmt1*, *dnmt3aa*, *dnmt3ab* and *dnmt3bb.1* from gastrula to hatching revealed that the transcript abundance of *dnmt1* was not significantly modified. Transcripts for the *dnmt3aa* and *dnmt3ab* paralogs increased (*P* < 0.05) and *dnmt3bb.1* decreased significantly (*P* < 0.05) (Fig. 2 and Table 3) from gastrula to hatch.

**Table 3:** Summary of expression results for the four dntms in sole. Results obtained about gene expression patterns as determined by qPCR and WISH in embryos, larvae and juveniles and their regulation by 5-AzaC and temperature

	Expression patterns^a^				Regulation^b^	
	Embryos	Lecitotrophic larvae	WISH patterns in larvae	Juveniles	5-AzaC	Temp
*dnmt1*	--	↓↓	Brain, spinal cord, eyes, pharynx and intestine	Brain	↓	---
*dnmt3aa*	↑↑	↓	Somites, pronephric duct, brain, spinal cord, eyes, pharynx and intestine	Spleen and gills	↑	↑↑
*dnmt3ab*	↑	↑	Brain, eye layers, spinal cord and intestine	Heart and brain	--	↑
*dnmt3bb.1*	↓	↓	Brain, pectoral fin buds, pronephric duct, eyes, intestine, pharynx and branchial arches	Gills	--	---

^a^The increase (↑) or decrease (↓) of gene expression from gastrula to hatch in embryos or during lecitotrophic larval development is shown

^b^The up- (↑) or downregulation (↓) of gene expression after 5-AzaC treatments or in response to low temperature (16 °C) is shown

The arrows indicate if expression is differentially induced (↑ = or reduced (↓) for each factor and their number denote the intensity of the response

**Fig. 3:**
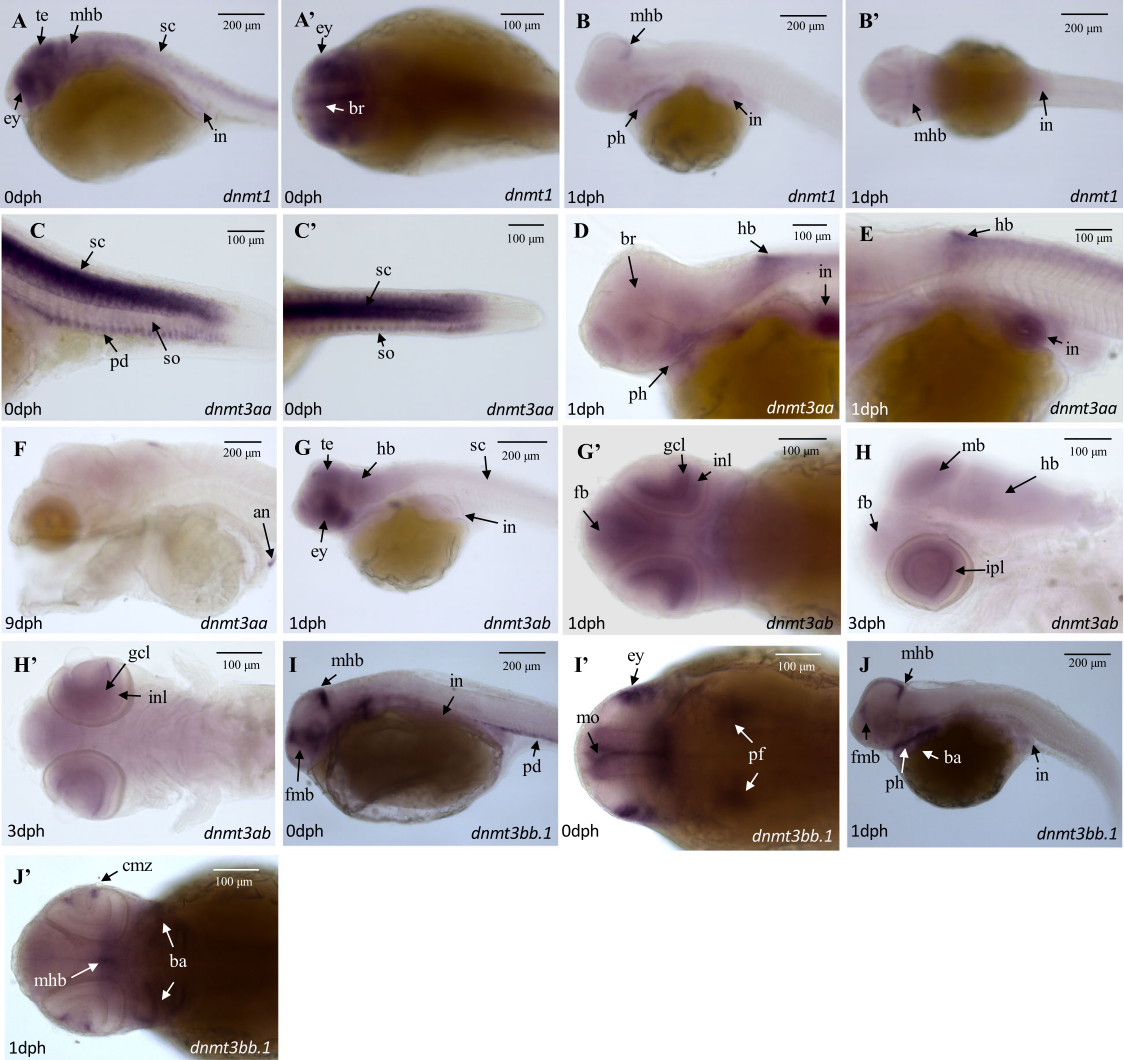
Temporal and spatial expression of methyltransferases. WISH signals for *dnmt1* (**A**, **B**)*, dnm3aa* (**C**-**F**)*, dnmt3ab* (**G**, **H**) and *dnmt3bb.1* (**I**, **J**) at different ages (0–9 dph) are shown. The ‘indicates the ventral orientation. Structures with an abundant signal are indicated with arrows: (an) anus; (ba) branchial arches; (br) brain; (cmz) ciliary marginal zone; (dm) dorsal midbrain; (ey) eye; (fb) forebrain; (fmb) forebrain-midbrain boundary; (gcl) ganglion cell layer; (hb) hindbrain; (inl) inner nuclear layer; (ipl) inner plexiform layer; (in) intestine; (mb) midbrain; (mhb) midbrain-hindbrain boundary; (mo) mouth; (pd) pronephric duct; (pf) pectoral fin buds; (ph) pharynx; (sc) spinal cord; (so) somite; (te) tectum. Scale bars are indicated (100 and 200 μm)

The spatio-temporal expression pattern of *dnmt1*, *dnmt3aa*, *dnmt3ab* and *dnmt3bb.1* in developing embryos was examined by whole-mount in situ hybridization (WISH; Fig. 3, Table 3 and Additional file 3). Expression for *dnmt1* was detected in the eyes, brain (mainly in the tectum), spinal cord and intestine at 0 dph (Fig. 3A, A’). At 1 dph, *dnmt1* was also detected in the posterior edge of the tectum closely to the midbrain-hindbrain boundary, pharynx and intestine (Fig. 3B, B‘). The *dnmt1* signal was weaker at 3 and 5 dph and was located mainly in the brain and spinal cord and at 9 dph a generalized low intensity signal was observed in the brain (Additional file 3). Expression of *dnmt3aa* was intense in the spinal cord, pronephric duct and somites at 0 dph (Fig. 3C, C‘) and by1 dph its expression was weaker in these organs and and it was also detected in the intestine, hindbrain and pharynx (Fig. 3D, E). As development progressed, *dnmt3aa* was mainly expressed in the brain, although by 9 dph it was only detected in the anus (Fig. 3F). The paralog *dnmt3ab* was predominantly observed in the hindbrain, midbrain (and tectum) and forebrain regions as well as in the inner nuclear layer, the inner plexiform layer and the ganglion cell layer of the developing eye at 1 and 3 dph (Fig. 3G,G‘,H,H’). A weak signal was also detected for *dnmt3ab* in the spinal cord and intestine up to 3 dph (Fig. 3G and Additional file 3). Expression of *dnmt3bb.1* at 0 dph was observed in the forebrain-midbrain and midbrain-hindbrain boundaries, in the pectoral fin buds, pronephric duct, eyes and a lower intensity signal was also detected in the intestine and mouth primordium (Fig. 3I, I‘). The *dnmt3bb.1* expression in 1 dph larvae was evident in the forebrain-midbrain and midbrain-hindbrain boundaries, pharynx, branchial arches, in the ciliary marginal zone of the eyes and in the intestine (Fig. 3J, J‘).

### Expression levels of dnmt1, dnmt3aa, dnmt3ab and dnmt3bb.1 in juvenile tissues

**Fig. 4:**
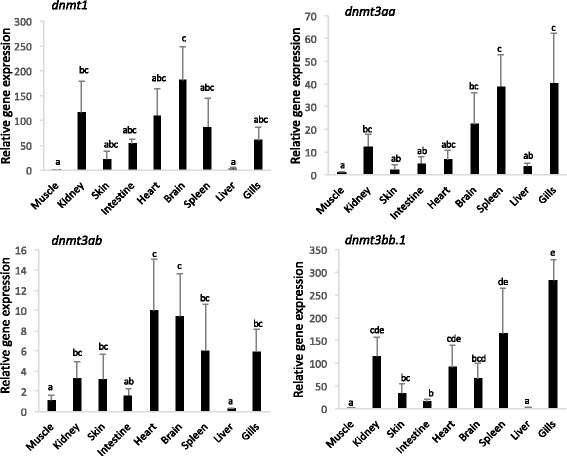
Relative expression levels of the four *dnmts* in sole juvenile tissues: muscle, kidney, skin, intestine, heart, brain, spleen, liver and gills. Data were expressed as the mean fold change (mean + SD, *n* = 4) from the calibrator group (muscle). Different letters denote statistically significant differences among tissues for each gene (*P* < 0.05)

The relative gene expression of the four methyltransferase coding genes was quantified by RT-qPCR in nine different tissues of juvenile sole (Fig. 4). The four genes were ubiquitously expressed in tissues although they were low abundance in liver (similar to the muscle that was used as the reference). In the case of *dnmt3aa* and *dnmt3bb.1* transcripts were most abundant in the kidney, spleen, brain and gills.

### Regulation of gene expression by 5-AzaCdR

**Fig. 5:**
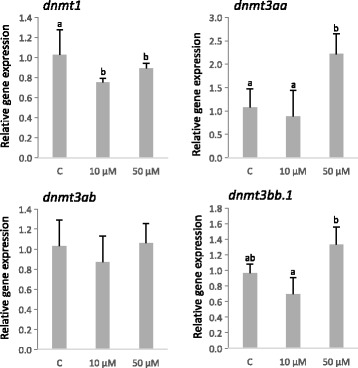
Relative expression levels of the four *dnmt* genes in sole larvae exposed to 5-AzaCdR (10 μM and 50 μM) for 24 h (at hatch). “C” denotes the control untreated group. Data are expressed as the mean fold change (mean + SD, *n* = 4) from the calibrator group (control). Different letters denote statistically significant differences among treatments for each gene (*P* < 0.05)

To explore the transcriptional regulation of the four *dnmt* transcripts by 5-AzaCdR, embryos in gastrula were exposed to 10 μM and 50 μM of the chemical for 24 h and the expression of *dnmts* quantified by RT-qPCR (Fig. 5). In larvae from eggs exposed to 50 μM 5-AzaCdR, *dnmt3aa* mRNA levels increased significantly (*P* < 0.05). In contrast, a significant reduction in *dnmt1* transcripts was detected in larvae exposed to 10 μM and 50 μM 5-AzaCdR relative to the control. No significant changes in the transcript abundance of *dnmt3ab* and *dnmt3bb.1* were detected relative to the control. WISH analysis did not reveal a change in the expression pattern between the control and 5-AzaCdR treated sole (see Additional file 4).

### Effect of temperature on expression levels of dnmt1, dnmt3aa, dnmt3ab and dnmt3bb.1 in larval lecithotrophic stages

**Fig. 6:**
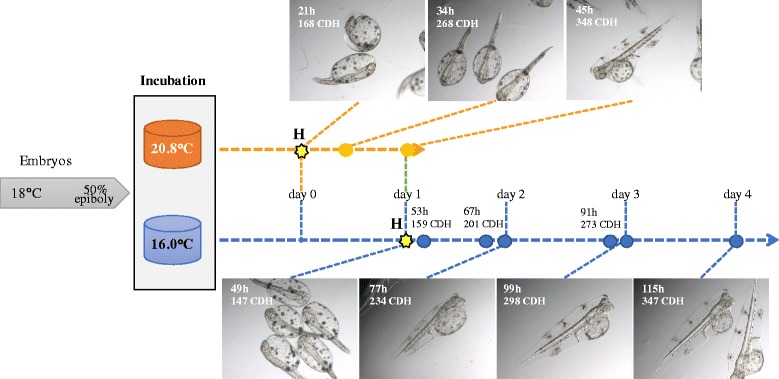
Experimental design for the evaluation of incubation temperatures during lecithotrophic stages in sole. The sampling points at 16 °C (*blue*) and 20.8 °C (*orange*) including the hours after incubation and the estimated CDH are indicated. A picture of the larvae sampled at each point is also shown. H denotes the hatching point

**Fig. 7:**
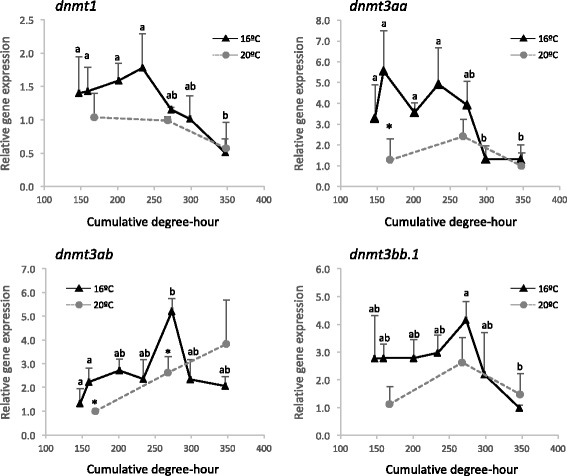
Expression levels of the four *dnmt* genes in lecithotrophic stages of sole incubated at 16 and 20 °C. Cumulative-degree hour was used to normalize larval developmental stages between different temperature groups. Data are expressed as the mean fold change (mean + SD, *n* = 3) from the calibrator group (173 CDH at 20 °C). *Asterisks* denote significant differences between different temperature treatments for the same CDH (*P* < 0.05). Different letters denote statistically significant differences due to age for each gene (*P* < 0.05)

To assess the effect of temperature on lecithotrophic larval stages (before mouth opening), embryos in gastrula were incubated at 20 °C or 16 °C for 45 and 115 h, respectively. The experimental design and sampling points are depicted in Fig. 6. To compensate for the delayed hatching and slower development and growth caused by lower water temperatures, cumulative-degree hours (CDH) were used to compare larvae at a similar developmental status. Comparison of the CDH values with larval total length, area and yolk sac length confirmed that this index gave a good estimate of developmental status (see Additional file 5). Two-way ANOVA showed that the expression of the four *dnmt* transcripts changed during development (Fig. 7). The *dnmt1*, *dnmt3aa* and *dnmt3bb.1* mRNA levels decreased with age whereas *dnmt3ab* expression levels peaked at 273 CDH. Moreover, temperature had a significant effect on the expression of *dnmt3aa* and *dnmt3ab* transcripts that were higher abundance at 16 °C relative to 20 °C. Incubation temperature did not significantly modify the expression of *dnmt3bb.1* and *dnmt1* transcripts. WISH was also carried out to determine how temperature affected *dnmt* transcript distribution. Temperature did not affect the expression pattern of the *dnmt1 and dnmt3* paralogs in sole larvae and the distribution was the same as described above (Additional file 5).

## Discussion

The Dnmt enzymes play an essential role in the epigenetic regulation of gene expression in early developmental stages of vertebrates. A previous analysis of the zebrafish genome identified up to eight DNA methyltransferase genes belonging to the Dnmt1 (1), Dnmt2 (1), and Dnmt3 (6) families [15] The Dnmt1 and 3 are involved in the maintenance and de novo methylation of DNA, respectively whereas Dnmt2, which was not analyzed in the present study, has been related with RNA methylation. Phylogenetic analysis indicated that Dnmt1 represents a single and highly conserved locus in teleosts and tetrapods suggesting its function has also been conserved during vertebrate evolution. In contrast, Dnmt3 is highly divergent from the Dnmt1 gene and is clustered into two main clades (referred to as *dnmt3a* and *dnmt3b*) and five subclades with *dnmt3aa*, *dnmt3ba* and *dnmt3bb.2* representing fish-specific gene lineages. The phylogenetic models in the present study are in general agreement with previous reports about Dnmt phylogeny [15–17]. However, the inclusion in the present study of all the identified Senegalese sole *dnmt3* genes and the teleost paralogs available from ENSEMBL refined the analysis of *dnmt3* subclades and fish-specific lineages.

The identification of at least five *dnmt* paralogs in the teleosts indicates that the diversification of the Dnmt3 lineage occurred early during vertebrate evolution during the proposed rounds of genome duplications, 1R, 2R and 3R (or the fish-specific whole duplication) that shaped their genomes [49]. Campos et al. [16] suggested that the *dnmt3* genes diverged after the 2R and 3R duplications, however the conservation of *dnmt3* in teleosts and the identification of three paralogs in spotted gar, a basal ray-finned fish [50], suggest that *dnmt3a* and *dnmt3b* clades might have arisen soon after the first genome duplication (1R). Later, before the tetrapod and teleost divergence, these lineages duplicated again (2R duplication) but only one paralog of each clade was retained in tetrapods. The 3R or fish-specific genome duplication gave rise to new paralogs but only five (two of which (*dnmt3aa* and *dnmt3bb.2*) are teleost-specific) were retained. Intriguingly, *dnmt3bb.1* shares higher sequence identity and phylogenetic proximity with *dnmt3ba* than *dnmt3bb.2* even though both *dnmt3bb*-like paralogs are arranged in tandem in the genome of some teleosts (e.g. stickleback, tetraodon, tilapia and zebrafish) suggesting they shared a common origin. The rapid divergence of *dnmt3b* paralogs in fish could be explained by the acquisition of an N-terminal calponin homology domain in *dnmt3ba* and *dnmt3bb.2* paralogs, as previously suggested, although little is known about its functional significance [15]. Moreover, lineage-specific duplications explain the occurrence of additional methyltransferases in some other species such as zebrafish that has two paralogs (*dnmt3* and *dnmt5*) within the subclade *dntm3bb.2*.

Methyltransferases are highly regulated during early development in order to tightly control expression cascades. Previous studies in *D. rerio* and *Carassius auratus* reported a higher abundance of *dnmt1* mRNA levels in early embryonic stages from cleavage to mid-blastula and a reduction close to hatch [51–53]. Our expression data reveal a similar expression profile for sole *dnmt1* during development although no significant differences were detected. The inverse correlation previously reported between *dnmt* gene expression and global DNA methylation levels has led to the suggestion that they may be maternal transcripts that direct methylation patterns in early stages [52]. An overview of *dnmt* expression patterns across teleosts suggests a conserved function in development. For example, *dnmt1* has a similar expression pattern in intestine, exocrine pancreas and retina of developing sole and zebrafish (72 h post-fertilization) [10]. A similar situation is observed with *dnmt3a* and *dnmt3b* paralogs. Both *dnnmt3aa* and *dnmt3ab* increase and *dnmt3bb.1* decrease in abundance in sole from gastrula to hatch. In zebrafish embryos RNA-seq data reveals that up to 24 hpf both *dnmt3a* paralogs increase in expression whereas the four *dnmt3b* genes gradually decrease [53, 54].

WISH in sole larvae revealed that *dnmt*’s had a distinct spatio-temporal expression pattern (Table 3) supporting the hypothesis that they play different roles in tissue differentiation. The tissue distribution of *dnmt3ab* and *dnmt3aa* during sole development suggests that subfunctionalization of these paralogs probably occurred. In contrast, the zebrafish, *dnmt3aa* and *dnmt3ab* had a similar, ubiquitous expression pattern in the brain, pharyngeal arches, pectoral fin buds, intestine, swim bladder and eye, although *dnmt3aa* expression was specific to the pronephric duct until 96 hpf [6, 14]. Interestingly, this is the first time that of *dnmt3aa* has been localized in the somites of a teleost fish and suggests it has a direct role in muscle differentiation and could be a putative candidate marker for epigenetic reprograming strategies with impact on growth and flesh quality in aquaculture. The expression pattern of sole *dnmt3bb.1* is similar to the ortholog *dnmt4* in zebrafish with a notably restricted expression in the lens epithelium and ciliary marginal zone [6, 14, 54] and a possible role in the differentiation and maintenance of the hematopoietic stem and progenitor cells lineages has been proposed [6, 55].

In addition to their role in early fish development, these *dnmt* genes have also been detected in varying abundance in adult tissues. For example, the *dnmt1* and *dnmt3b* paralogs were detected in a range of organs although they were mainly expressed in the gonads [17, 51, 53]. In contrast, both *dnmt3a* paralogs were ubiquitously expressed in adult tissues with a higher expression in brain and reduced expression in muscle [17, 53, 54]. The high expression of *Dnmt* genes in gonads has been linked to their role in maintenance of, and de novo methylation during gametogenesis [17]. In our study, the gonads were not evaluated but the four *dnmt* genes were expressed in brain and other organs with high levels of proliferation and involved in hematopoiesis such as the kidney, spleen or gills (Table 3). The higher expression of *dnmt genes* in other tissues than muscle suggests that *dnmt1*, *dnmt3a* paralogs and *dnmt3bb.1* play an active role in the regulation of cellular differentiation and methylation patterns in these adult tissues. Further research will be necessary to identify the precise role of each paralog.

The Dnmt inhibitors such as 5-azaC, and its deoxy analogue 5-azaCdR have been proposed as a tool to examine the epigenetic regulation of genes by environmental factors in fish [56]. Both inhibitors impose global DNA hypomethylation and DNA damage in cells although they can also exert drug-specific actions on cell viability, protein synthesis, the cell cycle and gene expression [57, 58]. In tumor cell lines, in vitro exposures to 5-azaCdR reduced *dnmt1* and *dnmt3b* mRNA expression and protein levels, and to a lesser extent *dnmt3a* [58–61]. The depletion in Dnmt1 was reported to be proteasome-mediated since it started even earlier than the incorporation of 5-azaCdR into DNA and the formation of aza-Dnmt-DNA adducts [62]. Although depletion of Dnmt’s is regularly observed in Dnmt inhibition studies, buffalo skin fibroblast cells exposed to 5-azaC decreased *dnmt1* but increased *dntm3a* and *dntm3b* mRNA levels at the highest 5-azaC concentrations [63]. Moreover, *dnmt1*, *dnmt3aa* and *dnmt3bb* transcript abundance increased at high doses (2 mM) of 5-azaC in medaka embryos indicating that protein depletion may be associated with transcriptional up-regulation of the genes [64]. In our study, *dntm1* was down-regulated and *dntm3aa* was up-regulated by high concentrations of 5-azaC (50 μM) suggesting that regulatory feedback may compensate for the inhibition of Dnmt activity. Since the *dnmt* genes exhibit variable spatio-temporal expression patterns in larvae, the impact of 5-azaC on larval reprogramming will likely depend on the timing and dose administered.

The inability of poikilotherms to regulate their temperature explains why changes in environmental temperature can profoundly influence the life cycle, including development of such animals. Temperature modulates larval phenotype by influencing metabolic rate, growth, energy stores, and sex determination and muscle development presumably through epigenetic mechanisms [19]. In this study, fertilized eggs were incubated at two temperatures within the physiological range at which spontaneous spawning occurs in the wild and in captivity [65]. An inverse relationship between the incubation temperature and development was observed as has previously been reported in several fish species including sole [38, 66–69]. A previous study in *S. senegalensis* using different incubation temperatures until hatch showed that temperature modified growth and muscle cellularity of benthic post-larvae [38]. Our study revealed that both *dnmt3aa* and *dnmt3ab* mRNAs were up-regulated in lecithotrophic larvae maintained at 16 °C relative to those maintained at 20 °C. The observation that somite-specific *dnmt3aa* was highly responsive to temperature may indicate that this paralog mediates the previously described modifications in methylation and myogenesis in sole cultured at different temperatures [37, 38]. In zebrafish, incubation temperatures also caused stage-dependent modifications in *dnmt3* paralog expression during embryonic development although they were not detected specifically in muscle [17]. Further work is required to identify modified gene expression linked to a specific phenotype but the present results on Dnmt have high potential for the aquaculture industry. Manipulation by temperature or other parameters of Dmnt represents a tool that can easily be used to enrich genetic breeding programs, and target muscle reprograming with implications for subsequent generations through the primordial germ cells [19, 32].

## Conclusions

This study identified *dnmt1* and five *dnmt3* paralogous genes in sole. Phylogenetic analysis revealed a high degree of conservation of the *dnmt* gene family in teleosts, with *dnmt3aa*, *dnmt3ba* and *dnmt3bb.2* resulting from fish-specific duplications. Clear spatio-temporal differences in *dnmt1*, *dnmt3aa*, *dnmt3ab* and *dnmt3bb.*1 expression profiles were detected in embryos, early larvae and juveniles. Moreover, expression of all *dnmt* genes decreased during larval development reinforcing their likely role in controlling cellular differentiation and development of larval tissues and organs. The Dnmt inhibitor 5-azaC modified expression of *dntm1* and *dntm3aa* and revealed as a useful tool to evaluate the impact of methylation during early sole development. Finally, the upregulation of *dnmt3aa*, the somite-specific isoform, and *dnmt3ab* at low incubation temperatures, identifies the paralogs as target Dnmt’s for epigenetic reprograming in this species.

## Additional files

Additional file 1: Sequence information used for phylogenetic analysis. The species, accession numbers (Ensembl^‡^, NCBI and SoleaDB^¥^), gene names and the abbreviations used in the tree are shown. (DOCX 128 kb)

Additional file 2: Phylogenetic relationships among the predicted amino acid sequences of *dnmt1* and *dnmt3* genes in Senegalese sole and other vertebrates (see Additional file 1) using the Maximum Likelihood method. The tree was rooted using *dnmt1* sequences. Only bootstrap values higher than 60% are indicated on each branch. The scale for branch length (2.0 substitutions/site) is shown below the tree. (JPEG 432 kb)

Additional file 3: Expression patterns of *dnmt1*, *dnmt3aa*, *dnmt3ab*, and *dnmt3bb*.1 in sole larvae. Lateral (panel A) and ventral (panel B) views for WISH analyses at 0, 1, 3, 5 and 9 dph are shown. (DOCX 46674 kb)

Additional file 4: WISH expression profiles of *dnmt1*(A), *dnmt3aa* (B)*, dnmt3ab* (C) and *dnmt3bb.1 (D)* in sole embryos exposed to 10 μM and 50 μM 5-AzaCdR concentrations for 24 h. Lateral and ventral views are shown in the top and bottom rows, respectively. Scale bars correspond to 200 μm. (DOCX 12609 kb)

Additional file 5: Biometric data and WISH analyses of larvae incubated at 16 and 20 °C during lecithotrophic stage. (DOCX 8164 kb)

## Abbreviations

5-AzaCdR: 5-aza-2′-deoxycytidine
an: Anus
BCIP: Bromo-chloro-indolyl-phosphate
bh: Branchial arches
br: Brain
CDH: Cumulative degree-hours
cmz: Ciliary marginal zone
dm: Dorsal midbrain
DMSO: Dimehtylsulfoxide
dph: Days post-hatch
ey: Eye
fb: Forebrain
fmb: Forebrain-midbrain boundary
glc: Ganglion cell layer
hb: Hindbrain
hpf: Hours post-fecundation
in: Intestine
inl: Inner nuclear layer
ipl: Inner plexiform layer
MAB: Malic acid buffer
MABTr: MAB + 0.1% TritonX-100
mb: Midbrain
mhb: Midbrain-hindbrain boundary
mo: Mouth
NBT: Nitro blue tetrazolium
pd.: Pronephric duct
pf: Pectoral fin buds
PFA: Paraformaldehyde
ph: Pharynx
sc: Spinal cord
so: Somite
te: Tectum
WISH: For whole-mount in situ hybridization
