# Supplementary material for: Phylogeny, expression patterns and regulation of DNA Methyltransferases in early development of the flatfish, Solea senegalensis
Source: BMC Dev Biol. 2017 Jul 17;17:11. doi: 10.1186/s12861-017-0154-0 (PMC5513168; doi:10.1186/s12861-017-0154-0)
Supplement: Supplementary file 1 — Sequence information used for phylogenetic analysis. The species, accession numbers (Ensembl‡, NCBI and SoleaDB¥), gene names and the abbreviations used in the tree are shown. (DOCX 128 kb) [file 12861_2017_154_MOESM1_ESM.docx]

***Additional file 1.*** Sequence information used for phylogenetic analysis. The species, accession numbers (Ensembl^‡^, NCBI and SoleaDB^¥^), gene names and the abbreviations used in the tree are shown.

|  | Species | Acc number | Gene name | Abbreviation |
| --- | --- | --- | --- | --- |
| *dnmt1* | *Solea senegalensis* | solea_v4.1_unigene57242^¥^ | *dnmt1* | Sse |
|  | *Astyanax mexicanus* | ENSAMXG00000012182^‡^ | *dnmt1* | Ame |
|  | *Danio rerio* | ENSDARG00000030756^‡^ | *dnmt1* | Dre |
|  | *Oreochromis niloticus* | ENSONIG00000001574^‡^ | *dnmt1* | Oni |
|  | *Gasterosteus aculeatus* | ENSGACG00000013741^‡^ | *dnmt1* | Gac |
|  | *Tetraodon nigroviridis* | ENSTNIG00000018567^‡^ | *dnmt1* | Tni |
|  | *Takifugu rubripes* | ENSTRUG00000008132^‡^ | *dnmt1* | Tru |
|  | *Oryzias latipes* | ENSORLG00000005582^‡^ | *dnmt1* | Ola |
|  | *Rattus norvegicus* | ENSRNOG00000039859^‡^ | *dnmt1* | Rno |
|  | *Gallus gallus* | ENSGALG00000028997^‡^ | *dnmt1* | Gga |
|  | *Xenopus_tropicalis* | ENSXETG00000022035^‡^ | *dnmt1* | Xtr |
| *dnmt3a* | *Solea senegalensis* | solea_v4.1_unigene37561^¥^  solea_v4.1_unigene339319^¥^  solea_v4.1_unigene511010^¥^ | *dnmt3ab* | Sse |
|  | *Danio rerio* | ENSDARG00000015566^‡^ | *dnmt3ab_dnmt6* | Dre_dnmt6 |
|  | *Oreochromis niloticus* | ENSONIG00000001050^‡^ | *dnmt3ab* | Oni |
|  | *Gasterosteus aculeatus* | ENSGACG00000011391^‡^ | *dnmt3ab* | Gac |
|  | *Tetraodon nigroviridis* | ENSTNIG00000019290^‡^ | *dnmt3ab* | Tni |
|  | *Takifugu rubripes* | ENSTRUG00000009738^‡^ | *dnmt3ab* | Tru |
|  | *Solea senegalensis* | solea_v4.1_unigene4300^¥^  solea_v4.1_unigene53289^¥^ solea_v4.1_unigene142077^¥^  solea_v4.1_unigene347645^¥^ | *dnmt3aa* | Sse |
|  | *Astyanax mexicanus* | ENSAMXP00000001710^‡^ | *dnmt3aa* | Ame |
|  | *Danio rerio* | ENSDARG00000005394^‡^ | *dnmt3aa _dnmt8* | Dre_dnmt8 |
|  | *Oreochromis niloticus* | ENSONIP00000006964^‡^ | *dnmt3aa* | Oni |
|  | *Gasterosteus aculeatus* | ENSGACG00000007097^‡^ | *dnmt3aa* | Gac |
|  | *Tetraodon nigroviridis* | ENSTNIP00000022331^‡^ | *dnmt3aa* | Tni |
|  | *Takifugu rubripes* | ENSTRUP00000009049^‡^ | *dnmt3aa* | Tru |
|  | *Oryzias latipes* | ENSORLP00000022087^‡^ | *dnmt3aa* | Ola |
|  | *Lepisosteus oculatus* | ENSLOCT00000020173^‡^ | *dnmt3a* | Loc |
|  | *Petromyzon marinus* | AAF05812 | *dnmt3a* | Pma |
|  | *Rattus norvegicus* | ENSRNOG00000026649^‡^ | *dnmt3a* | Rno |
|  | Gallus_gallus | ENSGALG00000003993^‡^ | *dnmt3a* | Gga |
|  | *Xenopus_tropicalis* | ENSXETG00000001232^‡^ | *dnmt3a* | Xtr |
| *dnmt3b* | *Solea senegalensis* | solea_v4.1_unigene12671^¥^ | *dnmt3bb.1* | Sse |
|  | *Astyanax mexicanus* | ENSAMXP00000020335^‡^ | *dnmt3bb.1* | Ame |
|  | *Danio rerio* | ENSDARG00000036791^‡^ | *dnmt3bb.1_dnmt4* | Dre_dnmt4 |
|  | *Oreochromis niloticus* | ENSONIP00000018686^‡^ | *dnmt3bb.1* | Oni |
|  | *Gasterosteus aculeatus* | ENSGACP00000013579^‡^ | *dnmt3bb.1* | Gac |
|  | *Tetraodon nigroviridis* | ENSTNIP00000009615^‡^ | *dnmt3bb.1* | Tni |
|  | *Takifugu rubripes* | ENSTRUP00000031152^‡^ | *dnmt3bb.1* | Tru |
|  | *Oryzias latipes* | ENSORLP00000025108^‡^ | *dnmt3bb.1* | Ola |
|  | *Latimeria chalumnae* | ENSLACP00000009510^‡^ | *dnmt3bb.1* | Lch |
|  | *Lepisosteus oculatus* | ENSLOCT00000006893^‡^ | *dnmt3bb.1* | Loc |
|  | *Solea senegalensis* | solea_v4.1_unigene187714^¥^ | *dnmt3bb.2* | Sse |
|  | *Danio rerio* | ENSDARG00000057830^‡^ | *dnmt3bb.2_dnmt3* | Dre_dnmt3 |
|  | *Danio rerio* | ENSDARG00000057863^‡^ | *dnmt3bb.2_dnmt5* | Dre_dnmt5 |
|  | *Oreochromis niloticus* | ENSONIP00000018679^‡^ | *dnmt3bb.2* | Oni |
|  | *Tetraodon nigroviridis* | ENSTNIP00000009617^‡^ | *dnmt3bb.2* | Gac |
|  | *Gasterosteus aculeatus* | ENSGACG00000010262^‡^ | *dnmt3bb.2* | Tni |
|  | *Takifugu rubripes* | ENSTRUP00000030962^‡^ | *dnmt3bb.2* | Tru |
|  | *Solea senegalensis* | solea_v4.1_unigene15160^¥^ | *dnmt3ba* | Sse |
|  | *Astyanax mexicanus* | ENSAMXP00000007814^‡^ | *dnmt3ba* | Ame |
|  | *Danio rerio* | ENSDARG00000052402^‡^ | *dnmt3ba_dnmt7* | Dre_dnmt7 |
|  | *Oreochromis niloticus* | ENSONIP00000021142^‡^ | *dnmt3ba* | Oni |
|  | *Gasterosteus aculeatus* | ENSGACG00000011949^‡^ | *dnmt3ba* | Gac |
|  | *Tetraodon nigroviridis* | ENSTNIP00000003297^‡^ | *dnmt3ba* | Tni |
|  | *Takifugu rubripes* | ENSTRUP00000012966^‡^ | *dnmt3ba* | Tru |
|  | *Oryzias latipes* | ENSORLP00000018521^‡^ | *dnmt3ba* | Ola |
|  | *Lepisosteus oculatus* | ENSLOCT00000006842^‡^ | *dnmt3ba* | Loc |
|  | *Rattus norvegicus* | ENSRNOG00000010625^‡^ | *dnmt3b* | Rno |
|  | *Homo sapiens* | AAF05812 | *dnmt3L* | Hsa |
