# Supplementary figures and images for: Phylogeny, expression patterns and regulation of DNA Methyltransferases in early development of the flatfish, Solea senegalensis
Source: BMC Dev Biol. 2017 Jul 17;17:11. doi: 10.1186/s12861-017-0154-0 (PMC5513168; doi:10.1186/s12861-017-0154-0)

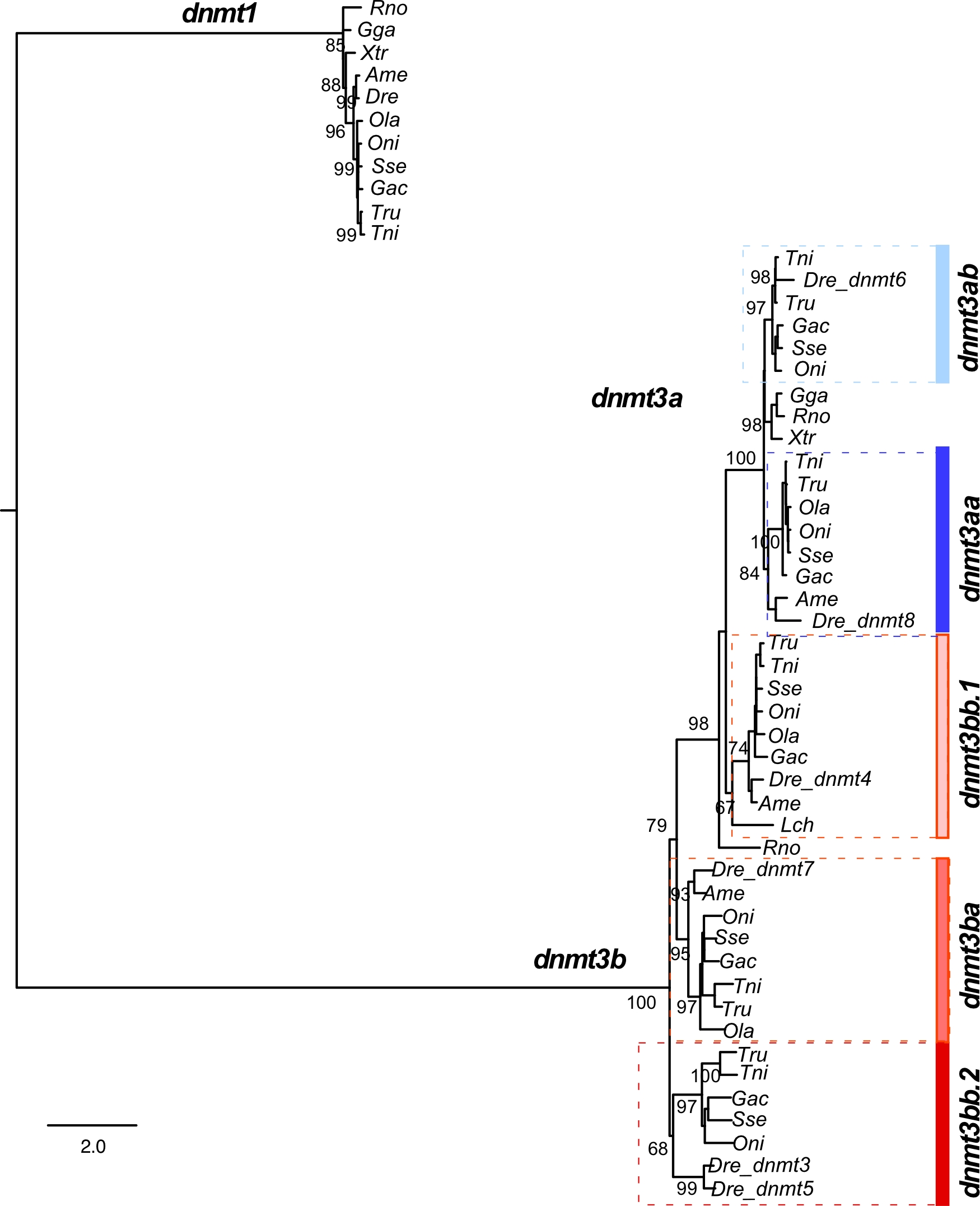

Supplement: Supplementary file 2 — Phylogenetic relationships among the predicted amino acid sequences of dnmt1 and dnmt3 genes in Senegalese sole and other vertebrates (see Additional file 1) using the Maximum Likelihood method. The tree was rooted using dnmt1 sequences. Only bootstrap values higher than 60% are indicated on each branch. The scale for branch length (2.0 substitutions/site) is shown below the tree. (JPEG 432 kb) [file 12861_2017_154_MOESM2_ESM.jpg]
