# Supplementary material for: Phylogeny, expression patterns and regulation of DNA Methyltransferases in early development of the flatfish, Solea senegalensis
Source: BMC Dev Biol. 2017 Jul 17;17:11. doi: 10.1186/s12861-017-0154-0 (PMC5513168; doi:10.1186/s12861-017-0154-0)
Supplement: Supplementary file 3 — Expression patterns of dnmt1, dnmt3aa, dnmt3ab, and dnmt3bb.1 in sole larvae. Lateral (panel A) and ventral (panel B) views for WISH analyses at 0, 1, 3, 5 and 9 dph are shown. (DOCX 46674 kb) [file 12861_2017_154_MOESM3_ESM.docx]

***Additional file 3.*** Expression patterns of *dnmt1*, *dnmt3aa, dnmt3ab*, and *dnmt3bb.1* in sole larvae. Lateral (panel A) and ventral (panel B) views for WISH analyses at 0, 1, 3, 5 and 9 dph are shown. A riboprobe from the sense strand was also used as a negative control (panel C). Structures with most expression signal are indicated by arrows: (an) anus; (ba) branchial arches; (br) brain; (cmz) ciliary marginal zone; (ey) eye; (fb) forebrain; (fmb) forebrain-midbrain boundary; (gcl) ganglion cell layer; (hb) hindbrain; (inl) inner nuclear layer; (ipl) inner plexiform layer; (in) intestine; (mb) midbrain; (mhb) midbrain-hindbrain boundary; (mo) mouth; (pd) pronephric duct; (pf) pectoral fin buds; (ph) pharynx; (sc) spinal cord; (so) somite; (te) tectum. Scale bars are represented (100 and 200 μm).

**A) *Lateral view***

**B) *Ventral view***

**C) *Negative controls (lateral view)***
