# Supplementary material for: Phylogeny, expression patterns and regulation of DNA Methyltransferases in early development of the flatfish, Solea senegalensis
Source: BMC Dev Biol. 2017 Jul 17;17:11. doi: 10.1186/s12861-017-0154-0 (PMC5513168; doi:10.1186/s12861-017-0154-0)
Supplement: Supplementary file 4 — WISH expression profiles of dnmt1(A), dnmt3aa (B), dnmt3ab (C) and dnmt3bb.1 (D) in sole embryos exposed to 10 μM and 50 μM 5-AzaCdR concentrations for 24 h. Lateral and ventral views are shown in the top and bottom rows, respectively. Scale bars correspond to 200 μm. (DOCX 12609 kb) [file 12861_2017_154_MOESM4_ESM.docx]

***Additional file 4.*** WISH expression profiles of *dnmt1*(A), *dnmt3aa* (B)*, dnmt3ab* (C) and *dnmt3bb.1 (D)* in sole embryos exposed to 10µM and 50µM 5-AzaCdR concentrations for 24h. Lateral and ventral views are shown in the top and bottom rows, respectively. Scale bars are represented (200 μm)

**A) *dnmt1***

**B) *dnmt3aa***

**C) *dnmt3ab***

**D) *dnmt3bb.1***
