# Supplementary material for: Phylogeny, expression patterns and regulation of DNA Methyltransferases in early development of the flatfish, Solea senegalensis
Source: BMC Dev Biol. 2017 Jul 17;17:11. doi: 10.1186/s12861-017-0154-0 (PMC5513168; doi:10.1186/s12861-017-0154-0)
Supplement: Supplementary file 5 — Biometric data and WISH analyses of larvae incubated at 16 and 20 °C during lecithotrophic stage. (DOCX 8164 kb) [file 12861_2017_154_MOESM5_ESM.docx]

***Supplementary file 5***. Biometric data and WISH analyses of larvae incubated at 16 and 20ºC during lecithotrophic stage. A) Biometric data; B-E) Expression pattern of the *dnmt1* (B), *dnmt3aa* (C), *dnmt3ab* (D) and *dnmt3bb.1* (E). Upper panel for WISH (named as .1) corresponds to larvae incubated at 16ºC 147 CDH, 20ºC 168 CDH and 16ºC 201 CDH; Lower panel for WISH (named as .2) corresponds to 16ºC 298.5 CDH, 20ºC 348 CDH and 16ºC 346.5 CDH. Lateral and ventral views within each WISH panel are shown. Scale bars are represented (100 and 200 μm).

**A)** Total length (TL), total area and Yolk Sac length (YSL) of larvae for each treatment (average hour temperature 16.0 and 20.8ºC). The hours (H) and corresponding CDH after treatments are indicated. Length measures are in mm and area in mm^2^.

| H | CDH | Treatment | TL | Area | YSL |
| --- | --- | --- | --- | --- | --- |
| 49.0 | 147 | 16ºC | 2.97±0.07 | 1.99±0.11 | 1.57±0.02 |
| 21.5 | 168 | 20ºC | 3.05±0.05 | 2.38±0.03 | 1.72±0.05 |
| 67.0 | 201 | 16ºC | 3.27±0.08 | 2.65±0.10 | 1.73±0.02 |
|  |  |  |  |  |  |
| 99.5 | 298.5 | 16ºC | 3.34±0.06 | 2.35±0.05 | 1.29±0.01 |
| 44.6 | 348 | 20ºC | 3.33±0.07 | 2.45±0.07 | 1.38±0.03 |
| 115.5 | 346.5 | 16ºC | 3.42±0.15 | 2.37±0.19 | 1.29±0.03 |

**B) Expression pattern of *dnmt1***

**B.1**. Expression of *dnmt1* at 147 (left), 168 (middle) and 201 (right) CDH

**B.2**. Expression of *dnmt1* at 298.5 (left), 348 (middle) and 346.5 (right) CDH

**C) Expression pattern of *dnmt3aa***

**C.1**. Expression of *dnmt3aa* at 147 (left), 168 (middle) and 201 (right) CDH

**C.2**. Expression of *dnmt3aa* at 298.5 (left), 348 (middle) and 346.5 (right) CDH


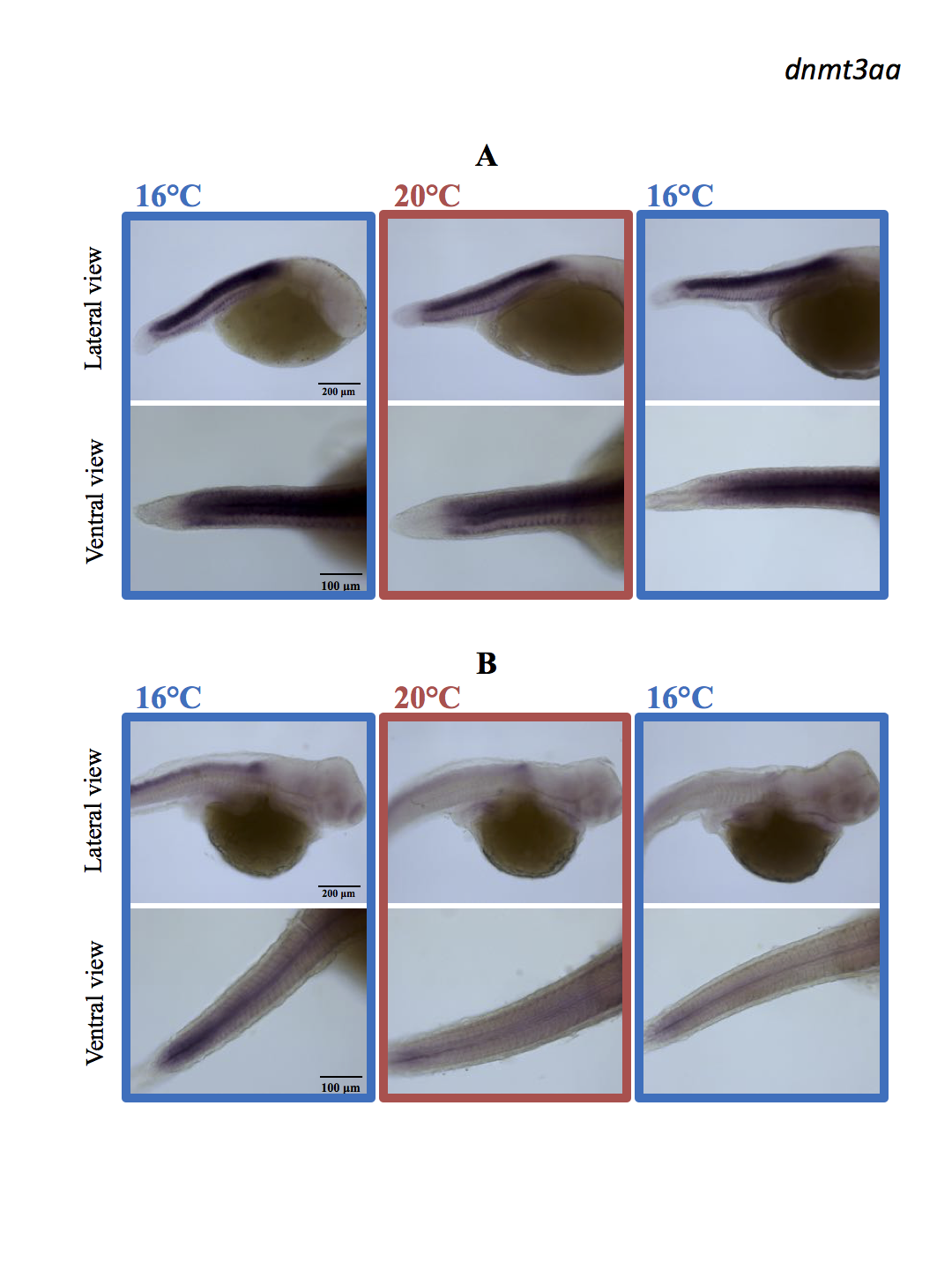


**D) Expression pattern of *dnmt3ab***

**D.1**. Expression of *dnmt3ab* at 147 (left), 168 (middle) and 201 (right) CDH

**D.2**. Expression of *dnmt3ab* at 298.5 (left), 348 (middle) and 346.5 (right) CDH

**E) Expression pattern of *dnmt3bb.1***

**E.1**. Expression of *dnmt3bb.1* at 147 (left), 168 (middle) and 201 (right) CDH

**E.2**. Expression of *dnmt3ab* at 298.5 (left), 348 (middle) and 346.5 (right) CDH
